# Activated phosphoinositide 3‐kinase delta syndrome: Pathogenesis, clinical manifestations, and treatment

**DOI:** 10.1002/pdi3.2504

**Published:** 2024-08-23

**Authors:** Ke Zhu, Qifan Li, Lingli Han, Jinqiao Sun

**Affiliations:** ^1^ Department of Clinical Immunology National Children Medical Center Children's Hospital of Fudan University Shanghai China

**Keywords:** activated phosphoinositide 3‐kinase delta syndrome, hematopoietic stem cell transplantation, inborn errors of immunity, targeted therapy

## Abstract

Activated phosphoinositide 3‐kinase delta syndrome (APDS) is an autosomal dominant inborn errors of immunity resulting from gain of function mutations in the *PIK3CD* gene or loss of function mutations in the *PIK3R1* gene. These mutations lead to hyperactivation of the phosphoinositide 3‐kinase/Akt/mammalian target of rapamycin (mTOR) signaling pathways, causing complex deficiencies in cellular and humoral immunity. APDS patients exhibit diverse clinical manifestations including recurrent respiratory infections, nonneoplastic lymphoproliferation, an increasing risk of malignancy, and autoimmune diseases. Neurodevelopmental abnormalities, short stature, failure to thrive, and psychological disorders are also observed. Management strategies for APDS involve antibiotic prophylaxis and immunoglobulin replacement therapy, immunosuppressive therapies, and hematopoietic stem cell transplantation for severe cases. Targeted therapies, such as the mTOR inhibitor sirolimus and the elective Phosphoinositide 3‐kinase delta inhibitor leniolisib, have emerged as promising options, demonstrating both safety and effectiveness. Continuous monitoring and further research are essential to optimize treatment strategies and understand the long‐term implications of these interventions. This review aims to summarize the pathogenesis, clinical manifestations, and treatment of APDS.

## 1 INTRODUCTION

Inborn errors of immunity (IEI) refers to a group of genetic disorders characterized by an increased susceptibility to infections, autoimmunity, and malignancy resulting from defects in the immune system. To date, there are over 480 genes that have been identified to cause various IEI.^1^ Activated phosphoinositide 3‐kinase delta syndrome (APDS) is an autosomal dominant IEI due to gain‐of‐function (GOF) mutations. It was first identified in 2013 by Lucas et al. in a study that described GOF mutations in the *PIK3CD* gene, which encodes the p110δ catalytic subunit of Phosphoinositide 3‐kinase delta (PI3Kδ), as the cause of a combined immunodeficiency syndrome.^2^ Later, patients with a highly similar clinical phenotype who did not carry *PIK3CD* mutations were found to possess loss‐of‐function (LOF) mutations in the *PIK3R1* gene, which encodes the p85α regulatory subunit of PI3Kδ.^3^ PI3Kδ, which is a catalytic subunit of the class I phosphoinositide 3‐kinase (PI3K) family, plays a crucial role in the immune system by regulating various cellular processes including cell growth, proliferation, differentiation, cytoskeletal organization, and intracellular trafficking.^4^

The GOF mutations in the *PIK3CD* gene and LOF mutations in the *PIK3R1* gene result in the hyperactivation of the PI3K/AKT/mammalian target of rapamycin (mTOR) signaling pathway in immune cells, leading to a complex deficiency of cellular and humoral immunity in APDS patients.^5^ Thus, patients with APDS may have immunodysregulatory and immunodeficient features including recurrent respiratory tract infections (RTIs), gastrointestinal symptoms, autoimmune disease, lymphoproliferation, and increasing risk of malignancy.^6^ As patients with *PIK3CD* GOF or *PIK3R1* LOF mutations both present abnormal PI3K signaling and similar clinical manifestations, the immune disorders arising from these two mutations have been designated as APDS1 and APDS2, respectively.

The therapy for APDS typically involves antibiotic prophylaxis and immunoglobulin replacement therapy (IRT) to reduce recurrent RTIs, immunosuppressive therapies to control autoinflammation and lymphoproliferation and hematopoietic stem cell transplantation (HSCT) for severe cases with life‐threatening complications. Targeted therapy, such as the mTOR inhibitor sirolimus and elective PI3Kδ inhibitor leniolisib, also demonstrated both safety and promising effect in the treatment of APDS.^7^

Given the increasing number of identified APDS patients and its heterogeneous manifestations, it is important to continuously refine diagnostic approaches, unravel underlying pathophysiological mechanisms, and optimize clinical care for individuals with APDS. This review aims to summarize the pathogenesis, clinical manifestations, and treatment of APDS. The goal is to provide a comprehensive understanding of APDS for the purpose of informing future research and advancing clinical management.

## 2 PATHOGENESIS

PI3Ks are a family of lipid kinases that regulate various cellular processes in mammalian cells including cell growth, proliferation, survival, cytoskeletal remodeling, and metabolism. They are categorized into three classes based on their structures and substrate specificity: class I, class II, and class III. Class IA PI3K molecules are composed of a p110 catalytic subunit and one of the five regulatory subunits. Specifically, the catalytic subunits, p110α, p110β, and p110δ, are encoded by *PIK3CA*, *PIK3CB*, and *PIK3CD* genes, respectively. While the former two isoforms are widely expressed across cell types, p110δ predominates in leukocytes.^8^ Regulatory subunits, including p85α, p55α, and p50α encoded by *PIK3R1*, along with p85β (encoded by *PIK3R2*) and p55γ (encoded by *PIK3R3*), modulate the activity of the catalytic unit by inhibiting its degradation and activity and recruits it to the plasma membrane.^9^

In immune cells, PI3Kδ which consists of p110δ and predominantly p85α is activated in response to extracellular stimuli through various cell receptors such as the T cell receptor (TCR), B cell receptor (BCR), toll‐like receptor, and cytokine receptors. The activated PI3Kδ then converts phosphatidylinositol‐4,5‐bisphosphate (PIP2) into phosphatidylinositol‐3,4,5‐trisphosphate (PIP3) as an important second messenger to phosphorylated AKT and activates the mTOR signaling pathway, which is crucial to the survival, differentiation, and function of T cells and B cells.^10^

In APDS, GOF mutations in *PIK3CD* and LOF mutations in both result in increased PI3kδ activity and hyperactivation of the PI3K/Akt/mTOR signaling pathway, resulting in T cells and B cells abnormalities. Hyperactive PI3kδ would disrupt the homeostasis of naïve T cells by promoting aerobic glycolysis, leading to their hyperproliferation and overactivation.^11^ Moreover, both CD4+ and CD8+ T cells were prone to activation‐induced cell death, which may also be related to the elevated proportions of activated/memory T cells.^2^ Additionally, while the number of T follicular helper cell was increased, their ability to help B cells differentiation was impaired.^12^ Together with B cell lymphopenia and class‐switch‐recombination defects (CSR‐Ds),^13^ antibody deficiencies and impaired humoral immune responses emerged, contributing to the manifestations of immunodeficiency and immunodysregulation of immune responses in patients with APDS.^14^

## 3 CLINICAL MANIFESTATIONS

### 3.1 Infective complications

Due to the immunodeficiency status, patients with APDS are susceptible to a diverse array of infections. Several cohort studies consistently reveal that most of APDS patients are reported to experience early onset, recurrent, and severe respiratory infections including sinusitis, nasopharyngitis, otitis media, mastoiditis, and pneumonia,^6^, ^15^ with *Haemophilus* influenzae and *Pneumococcus* pneumoniae being the most commonly identified respiratory pathogens. Moreover, infections with less common bacterial pathogens, such as *Staphylococcus aureus*, *Moraxella catarrhalis*, *Pseudomonas aeruginosa*, and *Klebsiella* species, were also reported.^13^ Bronchiectasis emerged as a common complication resulting from lung infections, impacting approximately 60% of patients with APDS1 and 8% of patients with APDS2.^14^ In addition to the infections of respiratory system, other sites of infections were also reported, including ocular infections presenting as conjunctivitis, orbital cellulitis and skin abscesses with *Staphylococcus aureus*, and gastrointestinal inflammation presenting as diarrhea with *Clostridioides difficile*, *Salmonella typhimurium*, and *Campylobacter* jejuni as well as osteomyelitis.^15^, ^16^

In addition, APDS is also associated with chronic nonresolving viral infections, with herpesvirus infections being the most common group induced by the Epstein–Barr virus (EBV), herpes simplex virus, cytomegalovirus, and varicella‐zoster virus, and associated with lymphoadenopathy.^17^, ^18^ Other infections by syncytial respiratory virus, pox virus, and papilloma virus infections were also reported for both types of APDS.^19^

### 3.2 Nonneoplastic lymphoproliferation

Activation of PI3Kδ signaling is also thought to trigger abnormal white blood cell proliferation, leading to lymphadenopathy and nodular lymphoid hyperplasia in about 75% patients with APDS and commonly presenting as hepatomegaly and splenomegaly.^6^ These enlargements, typically originating in childhood, tend to persist or recur especially at sites of infection. Moreover, nodular lymphoid infiltration becomes evident in various anatomical regions including the gastrointestinal tract, ear, nose, and throat. These infiltrations contribute to the clinical complexity of APDS, further exacerbating the challenges faced by affected individuals. Histological examination of lymph nodes showed atypical follicular hyperplasia characterized by the absence or attenuation of follicular mantle zones. Germinal centers were often disrupted and partially obliterated by numerous T cells, many of which expressed programmed cell death protein 1 (PD‐1), CD57, or both.^20^ Aggregation of monocytotic B cells were found in parasinusoidal space. These changes are generally nonneoplastic but would lead to various complications depending on the site of involvement. In addition, increased B cell proliferation is a risk factor for patients with compromised immune systems to develop lymphoma.^21^

### 3.3 Malignancy

Approximately, 13% of APDS patients were diagnosed with lymphoma including both Hodgkin and non‐Hodgkin's lymphoma.^6^ Intriguingly, a considerable proportion of these lymphoma cases exhibit an association with EBV infection. APDS patients were more susceptibility to malignancy maybe due to the inherent defects in lymphocytes and persistent stimulation of the lymphoid system by infections and autoimmune disorders.^22^

### 3.4 Autoimmune diseases

APDS is associated with an increased risk of autoimmune diseases. The persistent activation of the immune system and dysregulation of the immune system in APDS can lead to the production of autoantibodies, which target the body's own tissues and cells, resulting in autoimmune reactions.^23^ The precise mechanisms underlying the association between APDS and autoimmune diseases are complex and not fully understood, but they are thought to involve the impact of PI3Kδ signaling on immune cell function and regulation. Studies using a PI3Kδ E1020K mouse model suggest that polyclonal and self‐reactive B cells expand while number of antigen‐specific clone decreased.^24^ Chronic EBV infection, which promotes widespread B‐cell proliferation, also contributed to increasing risk of autoimmunity in APDS patients.^25^ Common autoimmune manifestations observed in APDS patients include cytopenias with autoimmune hemolytic anemia and immune thrombocytopenia being the most frequent autoimmune complications, arthritis, autoimmune enteropathy, chronic arthritis, autoimmune hepatitis, and chronic eczema.^26^ While autoimmune phenomena are common in patients with APDS, the frequencies can vary, and in some cases, they may be absent.^27^

### 3.5 Other complications

Neurodevelopmental abnormalities, such as speech delay, learning disabilities, and global developmental delay, have been reported in APDS patients, which were prevalent in those with APDS2 mutations.^28^, ^29^ APDS patients would also experience short stature and a general failure to thrive, which may result from recurrent infections and gastrointestinal inflammation. Other conditions including syndromic features, liver cysts, polycystic kidneys, and psychological disorders including depression, anxiety, and attention deficit have also been reported.^26^, ^30^

## 4 THERAPEUTIC OPTIONS

### 4.1 Antibiotic prophylaxis

Antibiotic prophylaxis is often considered as a part of the management strategy for patients with APDS. Since APDS patients are prone to recurrent infections due to immunodeficiency, antibiotic prophylaxis aims to prevent or reduce the frequency of bacterial, viral, or fungal infections. In two APDS cohorts, around 60% of patients received antibiotic prophylaxis.^6^, ^26^ Similar to those used in patients with antibody defects, the most commonly used regiments are trimethoprim/sulfamethoxazole or azithromycin.^31^ While using antibiotic prophylaxis alone may be sufficient in few patients, the majority required IRT. The combination of antibiotic prophylaxis with IRT is especially crucial for patients with RTIs and the established bronchiectasis to reduce the frequency of RTI.^14^ However, it was still insufficient in dealing with chronic viral infections and preventing lung damage. APDS patients experiencing chronic viral infections and progressive bronchiectasis were still reported despite undergoing IRT.^6^, ^26^

### 4.2 Immunoglobulin replacement therapy

Most patients with APDS exhibit various antibody defects, such as IgG subclass deficiencies, leading to ineffective vaccine responses and thus predispose to recurrent RTI. IRT is a key and effective treatment approach for these patients, administered either intravenous immunoglobulin (IVIG) or subcutaneously (SCIG). IRT is commonly used not only for APDS but also for other antibody deficiencies. For patients without bronchiectasis symptoms, IVIG is typically prescribed at a dose of 0.4 g/kg/month, with an increase to 0.6 g/kg/month in the presence of bronchiectasis.^32^, ^33^ This treatment has demonstrated efficacy in reducing RTIs. However, its effectiveness in preventing herpes infections, autoimmune disorders, and lymphoproliferation associated with APDS has not been clearly established.^34^ Further research is needed to determine the comprehensive impact of IRT on these specific aspects of the condition.

### 4.3 Hematopoietic stem cell transplantation

APDS may manifest or progress into a severe combined immunodeficiency, resulting in substantial morbidity and a higher risk of early mortality. Allogeneic HSCT is generally considered for patients with severe forms of APDS who experience life‐threatening complications, lymphoma, recurrent severe infections, or autoimmune manifestations that are unresponsive to conventional therapies. Frequent complications of HSCT included graft‐versus‐host disease (GvHD) and reduced or diminishing donor‐type chimerism.^35^

Nademi et al. presented 11 patients with APDS from seven pediatric centers who underwent HSCT due to severe clinical manifestations or poor clinical improvement following treatment with immunosuppressants or IVIG therapy. Of these patients, two died from viral infections after transplantation, one who had low chimerism, and eight were successfully transplanted and no longer required further immunosuppressive or IVIG therapy.^36^ Okano et al. present findings on the results of 23 patients diagnosed with APDS, among whom nine underwent HSCT due to a severe clinical course. Out of the nine patients, seven survived after frequent adverse complications and engraftment failure, with most symptoms showing improvement.^37^ In another cohort of 36 patients with APDS, 12 received HSCT and were all alive.^38^

An international retrospective study of 57 patients with APDS revealed that the 2‐year overall survival rate of HSCT reached 86%, with no difference in survival based on the donor type. The study also found that post‐HSCT use of mTOR inhibitors might benefit residual host cells, leading to graft instability and reduced transplantation success rates.^39^

These findings suggested that life‐threatening events, including severe infections caused by combined immunodeficiency, could be indicators of HSCT for patients with APDS but transplantation‐related complications including engraftment failure should be closely monitored.^36^ HSCT outcomes are also found to be more favorable in younger patients, consistent with observations in several other IEI.^40^ Nevertheless, the unpredictable nature of the disease course in young children introduces a potential risk, as there is a chance of subjecting patients with potentially mild conditions to the inherent risks associated with HSCT.

To date, no long‐term sequelae have been revealed with follow‐up periods ranging from 8 months to 16 years. However, uncertainties persist regarding the risks and advantages of HSCT in individuals with severe lung conditions. A more extensive follow‐up involving a larger cohort is necessary to determine the role of HSCT in addressing APDS.

### 4.4 Immunosuppressive therapies

Immunosuppressive therapies have been used to dampen autoinflammation and lymphoproliferation in patients with APDS. The B‐cell‐depleting antibody rituximab has been effectively employed in APDS patients to address hemolysis and lymphoproliferation. However, its use consistently results in significant and enduring hypogammaglobulinemia. Cytopenias, as the most common autoimmune disease in patients with APDS, can be controlled by steroids, rituximab, and splenectomy.^41^ Elgizouli et al. reported positive clinical outcomes using prednisolone and maintenance mesalazine to treat inflammatory bowel disease in APDS1.^34^ In cases involving sclerosing cholangitis, a severe complication of APDS, liver transplantation has been successfully performed, indicating that certain conditions associated with APDS may necessitate more invasive interventions to ensure optimal patient outcomes.^42^ Rituximab, specifically, emerges as a valuable component in managing nonneoplastic lymphoproliferation associated with APDS, encompassing conditions such as lymphadenopathy and hepatosplenomegaly.^41^

### 4.5 Targeted therapy

Sirolimus, also known by its trade name rapamycin, is a mTOR inhibitor. mTOR, a downstream protein kinase of the PI3K‐AKT pathway, plays an important role in T cell metabolism and acts as a key regulator in cell differentiation and immune response.^3^ Case series analysis have shown that rapamycin was effective to reduce hepatosplenomegaly and lymphadenopathy, restore T cell proliferation, and address nonneoplastic lymphoproliferation.^28^ However, its efficacy is less pronounced for cytopenias and gastrointestinal symptoms. Additionally, sirolimus has shown the benefit of decreasing the need for corticosteroids and enhancing lymphocyte subsets even at lower drug levels.^43^ Immunomodulation using rapamycin is more effective with fewer side effects when compared to the long‐term use of immunosuppressants such as corticosteroids. However, rapamycin does come with its set of side effects including symptoms resembling diabetes and lung toxicity. This is attributed to its target, mTOR, being not only implicated in BCR and TCR signaling via PI3Kδ but also in the insulin and growth hormone signaling pathways.^44^ Thus, it is important to assess the risk of hyperglycemia for patients undergoing long‐term rapamycin therapy. In conclusion, rapamycin proves to be beneficial for certain APDS patients; however, its use is limited by the requirement for continuous monitoring of trough levels and its limited effectiveness in addressing cytopenias, enteropathy, and infections.

The identification of the etiology of APDS and the causal involvement of mutations activating PI3Kδ have presented a new avenue for a unique and targeted treatment approach utilizing selective PI3Kδ inhibitors. This category of medications has been designed not only for cancer therapy but also for managing inflammatory conditions such as rheumatoid arthritis, asthma, and chronic obstructive pulmonary disease.^45^, ^46^ Leniolisib, an oral inhibitor targeting PI3Kδ, demonstrated promising outcomes in a randomized, placebo‐controlled phase III trial (NCT02435173).^47^ In the trial, Rao et al. presented efficacy data from six APDS patients who participated in a 12‐week dose‐escalation study of oral leniolisib administered twice daily. The drug was well tolerated and leniolisib led to a reduction in lymph node and spleen volumes, normalization of mean IgM levels, an increase in the number of naïve B cells, and a decrease in transitional B cells. The most noticeable normalization of immunophenotypes occurred in the final 4‐week dosing period. The study has progressed to a long‐term treatment arm, with patients receiving 70 mg leniolisib twice daily for over 9 months, and no significant adverse events have been reported.^48^ Thus, leniolisib has the potential to become a standard treatment for APDS patients aged 12 years and older.

The inhaled PI3Kδ inhibitor, nemiralisib, is also currently being studied in APDS. Nemiralisib exhibited positive outcomes in an animal model, reducing mortality in *Staphylococcus* pneumoniae‐infected mice with p110d^E1020K^.^49^ However, an open‐label trial involving five human subjects treated with nemiralisib over 3 months demonstrated that nemiralisib had an acceptable safety and tolerability profile, with cough being the most common adverse event, and no severe adverse events reported during the study.

However, the trial unveiled certain limitations. Despite the apparent safety, there were no substantial changes in blood inflammatory markers or lymphocytes. Furthermore, the study failed to provide convincing evidence of target engagement in the lung following inhaled dosing, and no downstream effects were observed in either the lung or blood compartments.^50^ The PI3K pathway is highly complex and may vary among different patients. Therefore, assessing the impact of PI3Kδ inhibitors at various points may be crucial for optimizing patient outcomes and further research is needed in this regard.

## 5 CONCLUSION

In recent decades, our comprehension of APDS has significantly advanced, which positions us to identify a previously uncharacterized cohort of patients in the future. As the spectrum of clinical manifestations varied in patients from mild forms to severe life‐threatening manifestations, the therapeutic options may also vary from mere observation to HSCT and now the promising targeted therapies. The emergence of targeted therapies, including the mTOR inhibitor sirolimus and PI3Kδ inhibitor leniolisib, presents a novel and promising avenue in APDS treatment. They demonstrated both safety and a promising effect. Despite these advancements, comprehensive studies are essential to evaluate the sustained efficacy, safety profiles, and potential adverse effects of targeted therapies in APDS patients over extended periods. This ongoing research will not only contribute to refining treatment guidelines but will also aid in identifying optimal therapeutic strategies for different manifestations and severities of APDS.

## AUTHOR CONTRIBUTIONS

Ke Zhu and Qifan Li collected data and drafted the manuscript. Lingli Han and Jinqiao Sun reviewed and revised the manuscript.

## CONFLICT OF INTEREST STATEMENT

No financial or nonfinancial benefits have been received or will be received from any party related directly or indirectly to the subject of this article.

## ETHICS STATEMENT

None.

## Data Availability

Data sharing is not applicable to this article as no new data were created or analyzed in this study.
